# Anabolic Factors and Myokines Improve Differentiation of Human Embryonic Stem Cell Derived Skeletal Muscle Cells

**DOI:** 10.3390/cells11060963

**Published:** 2022-03-11

**Authors:** Travis Ruan, Dylan Harney, Yen Chin Koay, Lipin Loo, Mark Larance, Leslie Caron

**Affiliations:** 1 Dr. John and Anne Chong Lab for Functional Genomics, Charles Perkins Centre, School of Life and Environmental Sciences, The University of Sydney, Sydney, NSW 2006, Australia; trua9904@uni.sydney.edu.au (T.R.); lipin.loo@sydney.edu.au (L.L.); 2 Larance Laboratory, Charles Perkins Centre, School of Life and Environmental Sciences, The University of Sydney, Sydney, NSW 2006, Australia; dylan.harney@sydney.edu.au (D.H.); mark.larance@sydney.edu.au (M.L.); 3 Cardiometabolic Disease Group, Heart Research Institute, Charles Perkins Centre, School of Life and Environmental Sciences, The University of Sydney, Sydney, NSW 2006, Australia; yen.koay@hri.org.au; 4 MMG, Marseille Medical Genetics, Aix Marseille Univ, INSERM U1251, 13005 Marseille, France

**Keywords:** skeletal muscle, myotubes, myokines, human embryonic stem cell, myosin heavy chain

## Abstract

Skeletal muscle weakness is linked to many adverse health outcomes. Current research to identify new drugs has often been inconclusive due to lack of adequate cellular models. We previously developed a scalable monolayer system to differentiate human embryonic stem cells (hESCs) into mature skeletal muscle cells (SkMCs) within 26 days without cell sorting or genetic manipulation. Here, building on our previous work, we show that differentiation and fusion of myotubes can be further enhanced using the anabolic factors testosterone (T) and follistatin (F) in combination with a cocktail of myokines (C). Importantly, combined TFC treatment significantly enhanced both the hESC-SkMC fusion index and the expression levels of various skeletal muscle markers, including the motor protein myosin heavy chain (MyHC). Transcriptomic and proteomic analysis revealed oxidative phosphorylation as the most up-regulated pathway, and a significantly higher level of ATP and increased mitochondrial mass were also observed in TFC-treated hESC-SkMCs, suggesting enhanced energy metabolism is coupled with improved muscle differentiation. This cellular model will be a powerful tool for studying in vitro myogenesis and for drug discovery pertaining to further enhancing muscle development or treating muscle diseases.

## 1. Introduction

Skeletal muscle is the most abundant tissue in the human body, making up around 40% of total body weight. Skeletal muscle is essential for movement and metabolic health, and diseases of muscle function can arise due to genetic mutations; metabolic or neuromuscular dysfunctions; or natural aging [1,2]. Skeletal muscle disorders are linked to many adverse health outcomes, such as impaired mobility, falls, fractures, frailty, diminished quality of life and premature death [3]; and research to identify new drugs has often been inconclusive due to lack of adequate skeletal muscle models.

Due to inter-species differences, animal models and rodent cell lines (e.g., C2C12 myoblasts, L6) do not accurately reflect all aspects of human muscle development [4,5]. Primary myoblasts obtained from patients’ biopsies have often been used for research but are limited in number, phenotypically diverse and have poor expandability, restricting their utility. Human pluripotent stem cells (hPSCs), on the other hand, offer a major advantage for studying human skeletal muscle development. Their capacity to proliferate indefinitely and differentiate into most cell types of the human body make them an excellent and renewable source of human skeletal muscle cells (SkMCs). In recent years, human induced pluripotent stem cell (hiPSC) and human embryonic stem cell (hESC)-derived models from patients with muscular diseases have become useful tools for modeling a large spectrum of inherited neuromuscular diseases [6,7,8]. While initially challenging and very inefficient, recently proposed protocols utilize small molecules to recapitulate the embryonic development of skeletal muscle [6,7,9,10,11]. However, the majority of these protocols give rise to heterogeneous cell populations, and their reproducibility with multiple hPSC lines has proven a challenge. The methods described by Chal et al. and Shelton et al., for example, have been shown to give rise to a mixed population of myogenic and non-myogenic cells, including different types of neurons and other unidentified cell types [12,13]. This cell variability may affect the effectiveness of differentiation towards SkMCs and the subsequent downstream analysis.

We previously developed a monolayer system to efficiently and reproducibly differentiate hPSCs into a pure and functional population of SkMCs [6]. This protocol produced myosin heavy chain (MyHC)-positive SkMCs within 26 days that can be expanded in large quantities. The protocol has great potential for the study of human skeletal muscle development and diseases, and drug screening. It can be adapted to 3D systems and generates contractile myofibers when combined with a biocompatible hydrgel for the generation of artificial human muscle [14]. Our system has been efficiently used with over 40 hPSC lines and is now used extensively in multiple laboratories for the modelling of muscular dystrophies or insulin resistance/type 2 diabetes [15,16,17,18]. However, the myotubes remain thin and contain few nuclei, suggesting that the last stage of the differentiation can be further improved.

To improve our skeletal muscle differentiation protocol, we investigated the effects of the well-known anabolic compounds testosterone (T) and follistatin (F), and the effects of a cocktail of myokines (C), on hESC-SkMCs. While these growth factors and hormones have been extensively studied in vivo or in primary SkMCs, their combinatorial effect on hPSCs’ differentiation into SkMCs has not be reported. Here we show that a combination treatment using T, F and C (TFC) had a significant and synergistic effect on hESC-SkMC terminal differentiation and enhanced both the hESC-SkMC fusion index and the expression levels of various skeletal muscle markers. Transcriptomic and proteomic analysis revealed oxidative phosphorylation as the most up-regulated pathway, suggesting these cells also have a greater capacity for energy metabolism.

## 2. Materials and Methods

### 2.1. Human Stem Cell Culture

GENEA002 and GENEA016 cell lines were obtained from Genea Biocells Ltd. (Sydney, Australia) and were previously described in [19,20]. The ATCC-BXS0116 hiPSC line was used in this study (ATCC catalog number ACS-1030). The H9 cell line was obtained from the WiCell institute. 

GENEA016, GENEA002, H9 hESC and ATCC-hiPSC were cultured on Matrigel^®^ (Corning) coated plates in mTeSR^TM^1 (StemCell Technologies), Vancouver, BC, Canada supplemented with 0.5% penicillin–streptomycin (ThermoFisher Scientific, Waltham, MA, USA). Cells were grown in a 37 °C incubator with 10% O_2_ and 5% CO_2_ and passaged every 3–4 days as needed.

### 2.2. Skeletal Muscle Cell Differentiation

hPSC were differentiated into skeletal muscle cells following the protocol described in Caron et al. [6] in a 10% O_2_ and 5% CO_2_ controlled incubator. Briefly, cells were seeded at a density of 2500 cells per cm^2^ onto collagen type I (Sigma-Aldrich, St. Louis, MO, USA) coated plates in Skeletal Muscle Induction Media (5% horse serum, 3 μM CHIR99021, 2 μM ALK5 inhibitor, 10 ng/mL hr-EGF, 10 μg/mL insulin, 0.4 μg/mL dexamethasone and 200 μM ascorbic acid) for 10 days with medium change every 2–3 days. At day 10, cells were dissociated and re-plated onto new collagen type I-coated plates at 2500 cells per cm^2^ in Skeletal Muscle Myoblast Media (5% horse serum, 10 μg/mL insulin, 10 ng/mL hr-EGF, 20 ng/mL hr-HGF, 10 ng/mL hr-PDGF, 20 ng/mL hr-bGFG, 20 μg/mL oncostatin, 10 ng/mL IGF-1, 2 μM SB431542 and 200 μM ascorbic acid) with medium change every 2–3 days. At day 20, cells were switched to Skeletal Muscle Myotube Media (10 μg/mL insulin, 20 μg/mL oncostatin, 50 nM necrosulfonamide and 200 μM ascorbic acid) with medium change every 2–3 days. The Skeletal Muscle Myotube Media were used for the culturing and differentiation of non-treated myotubes (NTC). For TFC treatment, cells were cultured in Skeletal Muscle Myotube Media with the addition of 400 ng/mL testosterone, 300 ng/mL follistatin, 1 mM creatine, 150 ng/mL Il6, 20 ng/mL Il4, 20 ng/mL BDNF and 25 ng/mL VEGF (Supplementary Table S2). For all myotube differentiation experiments performed in this manuscript, cells were differentiated (with or without treatment) for 96 h before analysis. 

### 2.3. Immunofluorescence Staining

Cells were washed with PBS and fixed with 4% formaldehyde for 15 min at room temperature, followed by three PBS washes and blocking in 1% BSA/PBS for 30 min. Cells were stained with primary antibody in permeabilization buffer (1% BSA and 0.1% Triton-X 100 in PBS) and incubated overnight in 4 °C. On the next day, cells were washed three times with PBS and incubated with 1:500 Hoechst and appropriate secondary antibody in permeabilization buffer for one hour at room temperature. The cells were washed with PBS three times before imaging. 

### 2.4. High Content Imaging and Analysis

Plates were imaged on an Opera Phenix^TM^ High-Content Screening System (Perkin Elmer, Waltham, MA, USA) at 20× magnification, and 49 fields (7 × 7) were imaged on every well. This imaging depth captures approximately 90% of the total area of an individual well. Image analysis was performed with Opera Phenix^TM^’s analysis software Harmony using a custom built myotube analysis pipeline. Briefly, MyHC was determined by MF20 signal, and a thresholding filter was applied to remove non-specific and background signal. The dimension of MF20 was therefore considered as indicative of MyHC size. Similarly, nuclei were identified by Hoechst signal followed by a threshold filtering to remove background noise. Fusion index is calculated as the number of nuclei within MyHC+ area divided the number of total nuclei.

### 2.5. Calcium Imaging

The calcium imaging protocol was adapted from [21]. Briefly, cells were loaded with FURA-2AM (Invitrogen, Waltham, MA, USA) in Hank’s Balanced Salt Solution (HBSS) for 30 min in the dark at 37 °C. Cells were washed three times with HBSS and maintained at 37 °C for 15 min prior to imaging on Nikon TI Live Cell Microscope (Nikon, Tokyo, Japan). Bath application of agonist (3 mM nicotine) was performed after 70 s of baseline recording. The fluorescence ratio (F340:F380) was extracted from cells using Nikon NIS-Element software and presented as normalized mean ± SEM.

### 2.6. ATP Determination Assay

ATP determination was performed using the ATP Determination Kit (A22066, Thermo Fisher, Waltham, MA, USA) following the manufacturer’s protocol. Eight different batches of myoblasts were differentiated separately and subsequently transferred to individual wells of a new plate; then, ATP determination was performed. Briefly, cells were lysed and supernatants incubated with recombinant firefly luciferase and its substrate D-luciferin. This assay is based on luciferase’s absolute requirement for ATP to produce light. Samples were read using a luminescence plate reader, and ATP level was normalized to protein level using the bicinchoninic acid protein assay.

### 2.7. Mitotracker^TM^ Deep Red Staining

Mitochondrial mass was determined using the Mitotracker^TM^ Deep Red FM fluorescent dye (M22426, Thermo Fisher, Waltham, MA, USA) following the manufacturer’s protocol. Briefly, cells were incubated with Mitotracker for 40 min in three concentrations (0/50/250 nM) to allow permeabilization and imaged using Opera Phenix^TM^. Images were analyzed using Opera Phenix^TM^’s analysis software Harmony using a custom built Mitotracker analysis pipeline.

### 2.8. RT-qPCR

Cells were collected from three different batches of D24 myotubes differentiated on different days. RNA were extracted using FavorPrep^TM^ Blood/Cultured Cell Total RNA Kit (Fisher Biotec, Wembley, Australia) and quantified using Qubit^TM^ RNA BR Assay Kit (Thermo Scientific, Waltham, MA, USA). cDNA was synthesized using iScript^TM^ Reverse Transcription Supermix for RT-qPCR (Bio-Rad, Hercules, CA, USA), and RT-qPCR was performed using the SYBR^TM^ Select Master Mix (Thermo Scientific) on LightCycler^®^ Instrument II (Roche, Basel, Switzerland). The full list of primers for RT-qPCR are available in Table S3.

### 2.9. RNASeq

All RNASeq experiments were performed in triplicate from three independent biological replicates. RNA was extracted and quantified as described above. RNA was treated with DNAse in solution using the On-Column DNase I Digestion Set (Sigma-Aldrich, St. Louis, MO, USA) and maintained with Ribosafe RNAse Inhibitor (Bioline, London, UK). Quality and quantity of RNA were assessed by Nanodrop (Thermo Scientific, Waltham, MA, USA), Qubit (Thermo Scientific, Waltham, MA, USA) and Bioanalyzer (Agilent, Santa Clara, CA, USA). Library preparation and sequencing were performed by Novogene. STAR aligner [22] was used for mapping sequence reads to the human genome (hg38 assembly), allowing up to three mismatches and retaining only reads that aligned with unique locations. Ensemble gene models [23] was used for quantifying gene expression from mapped reads using featureCounts [24], and genes that were lowly expressed (less than two samples with counts >10) were removed from subsequent analysis. Raw read counts were analyzed in RStudio using DESeq2, and differential expression was assessed [25]. Genes with adjusted *p*-values of <0.05 (Benjamani–Hochberg corrected) were assessed as differentially expressed.

### 2.10. Proteomics

All proteomics experiments were performed in triplicate from three independent biological replicates. The proteomics protocol was adapted from [21]. Cells were lysed in 4% sodium deoxycholate (SDC) buffer and heat inactivated for 10 min at 100 °C. Sonicated samples were quantified using a bicinchoninic acid protein assay (Thermo Scientific), following the manufacturer’s instructions, to determine protein concentration. Tandem mass spectrometry was carried out on a Q-Exactive Mass Spectrometer (Thermo Scientific). Raw data were processed using MaxQuant using human UniProt database. The LFQ intensity values (quantification of proteins) were log-transformed (base 2). We next filtered proteins, requiring all biological replicates to be quantified in at least one condition. After filtering, the missing values were then imputed using the tail imputation method with a Gaussian distribution of $N\left(\mu -\sigma *1.8,\;\sigma *0.3\right)$ as in [26]. The imputed data were next converted to ratios relative to the control condition and normalized using Combat [27] to remove additional unwanted variation. Differentially regulated proteins were determined using ANOVA test with an adjusted *p*-value of <0.05.

### 2.11. Metabolomics

All metabolomics experiments were performed in triplicate from three independent biological replicates. Cells were harvested and subjected to metabolite extraction as described in [28], with some minor modifications. Cells were washed twice with cold sodium chloride and scraped into a 500 µL 50% (*v*/*v*) methanol:water mixture containing internal standards of 10 mM phenylalanine-d_8_, valine-d_8_ and thymine-d_4_ on ice (4 °C). Then, 500 µL of chloroform was added to the extracts and the solutions vortexed. The aqueous phase was separated from the insoluble and organic layers by centrifugation at 16,000× *g*, 4 °C for 20 min. The upper aqueous phase was subjected to drying using SpeedVac Vacuum concentrator and resuspended in acetonitrile:methanol:formic acid (75:25:0.2; *v*/*v*/*v*, HPLC grade; Thermo Fisher Scientific) for HILIC-MS and in acetonitrile:methanol (75:25; *v*/*v* HPLC grade; Thermo Fisher Scientific) for AMIDE-MS. Every extraction condition was prepared in three biological replicates. Samples were analyzed by liquid chromatography–tandem mass spectrometry (LC-MS/MS).

LC-MS/MS analysis was performed using an Agilent Infinity 1260 LC coupled to an AB Sciex QTRAP 5500 MS. LC separation for AMIDE-MS method was achieved on a XBridge Amide column (4.6 mm × 100 mm, 3.5 umL Waters Australia) at ambient temperature using buffer A—95:5, *v*/*v*, water:acetonitrile containing ammonium hydroxide and ammonium acetate, both at 20 mM (pH 9.3)—and buffer B (100% acetonitrile). LC separation for HILIC-MS method was performed on Atlantis^®^ HILIC column (2.1 mm × 150 mm, 3 um; Waters Australia) using buffer A (water containing formic acid (0.1%) and ammonium formate (10 mM)) and buffer B (acetonitrile containing formic acid (0.1%)). 

All raw data files (Analyst software, version 1.6.2; AB Sciex, Foster City, CA, USA) were acquired and imported into Multi-Quant^TM^ 3.0 for MRM Q1/Q3 peak integration. 

### 2.12. Pathway Analysis

Pathway analysis of transcriptomic and proteomic data was performed on Ingenuity Pathway Analysis (IPA) using genes with adjusted *p*-values of <0.05 as input. Gene Set Enrichment Analysis (GSEA) was used to determine whether there were significant differences between treatments using default parameters. 

## 3. Results

### 3.1. hESCs Differentiate into Functional SkMCs In Vitro

**Figure 1:**
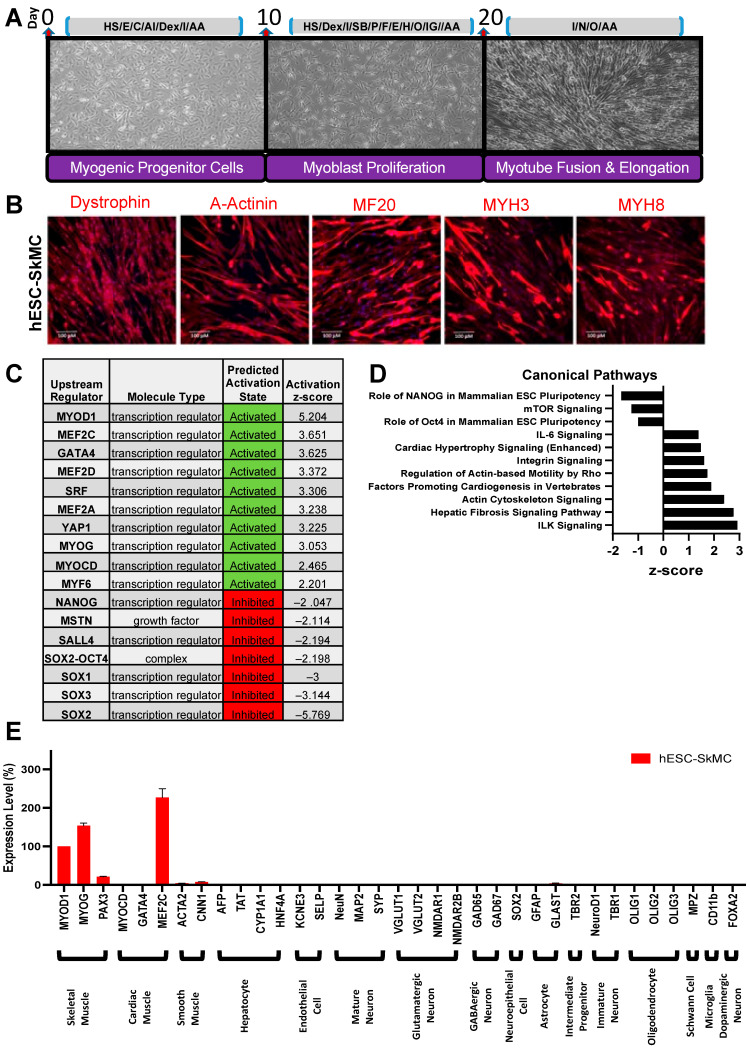
Skeletal muscle differentiation of hESCs. (**A**) Differentiation protocol for the derivation of SkMCs from hESCs. HS = horse serum, E = hr-EGF, C = CHIR99021, AI = ALK5 inhibitor, Dex = dexamethasone, AA = ascorbic acid, I = insulin, SB = SB431542, P = hr-PDGF, F = hr-FGFB, H = hr-HGF, O = oncostatin, IG = hr-IGF1, N = necrosulfonamide. (**B**) hESC-SkMCs expressed high levels of SkMC markers dystrophin, a-actinin, MF20 (MYH all isoforms) and embryonic (MYH3) and perinatal (MYH8) myosin. Scale bar = 100 μm. (**C**) IPA transcriptomic analysis of upstream regulators between hESCs and hESC-SkMCs and their activation status. (**D**) IPA transcriptomic analysis of differentially regulated canonical pathways in hESCs and hESC-SkMCs. (**E**) RNASeq data showing hESC-SkMCs express markers specific to skeletal muscle lineage. Shown are data pooled from 3 independent biological replicates.

We first differentiated GENEA016 hESCs into hESC-SkMCs by following the protocol described by Caron et al. [6], and confirmed the myogenic identity of these cells by immuno-staining for markers representative of each stage (Figure 1A,B and Supplementary Figure S1A–C). During myogenic lineage induction (days 1–10), cells stained positive for PAX3 and PAX7, two transcription factors known for their roles in the early phase of myogenesis [29] (Supplementary Figure S1A). After the second phase of differentiation (days 10–20), 58.2% of the cells were positive (by staining) for MyoD1 at D20, the master regulator of myogenesis indicative of myogenic lineage commitment [30] (Supplementary Figure S1B). After myoblasts elongation and fusion during the terminal phase of differentiation, 79.4% of myotubes were positive (by staining) for the skeletal muscle marker myogenin (Supplementary Figure S1C); and myotubes expressed the sarcomeric proteins dystrophin, alpha-Actinin and MyHC (Figure 1B). As previously found, MyHC (MF20 staining) was detected in 70% of differentiated hESC-SkMCs (D24) and not in pre-differentiated hESCs (D0) (Supplementary Figure S1D). hESC-SkMCs expressed MYH3 (embryonic) and MYH8 (perinatal), but not MYH1 or MYH2 (adult) isoforms (Supplementary Figure S1E), indicating a relatively immature phenotype [6].

We compared the transcriptomic and proteomic profiles of hESCs and hESC-SkMCs, and found an expression pattern associated with cell differentiation, including down-regulation of key pluripotency genes NANOG, POU5F1 and SOX2; and down-regulation of proliferation and cell cycle genes, including MKI67 and CDK1, at the protein level (Supplementary Figure S1F). CDK4 was up-regulated, and it has been shown to allow myogenic cells to recapture growth potential without compromising differentiation potential [31]. Additionally, we performed IPA (Log2FC +/−4, padj < 0.05) to identify the cascade of upstream transcriptional regulators that can explain the observed changes in gene expression. We assessed the expression levels of numerous key muscle and pluripotency transcriptional factors, and their activation z-scores suggest enhancements of muscle differentiation pathways and inhibition of pluripotency pathways consistent with in vitro muscle differentiation (Figure 1C). The core pluripotency network, including OCT4, NANOG and SOX2, was predicted as inhibited. Similarly, myostatin, a well-known myokine that inhibits myogenesis [32], was predicted as inhibited. In contrast, myogenesis transcription regulators, including all members of the myogenic regulatory factors (MRF) MyoD1, MYOG, MYF5 and MYF6 (also known as MRF4) were activated (Figure 1C, Supplementary File S1). These myogenic factors are involved in cell specification of the skeletal muscle lineage and are important in the generation of both developing and mature skeletal muscle [33]. These transcriptomic changes are associated with modulation of several signaling pathways (Supplementary File S2). Some examples include down-regulation of pathways involved in cell pluripotency and proliferation, and up-regulation of those that are involved in myocyte contractility (ILK Signaling) [34], cell–cell adhesion (Integrin Signaling) [35] and sarcomere integrity (Cardiac Hypertrophy Signaling) [36] (Figure 1D). To confirm the cell purity of our differentiation system, we evaluated the expression levels of specific markers of various cell types by RNAseq in our differentiated population relative to MyoD1 expression levels. Non-muscle markers were not expressed in hESC-SkMCs. This includes markers for several neuronal lineages and astrocytes, oligodendrocytes, hepatocytes and endothelial cells (Figure 1E). Cardiac and smooth muscle markers were also undetected in our hESC-SkMC, with the exception of MEF2C and CNN1. While MEF2C is a cardiac lineage marker, it is also expressed in skeletal muscle during development [8], which explains its presence in our hESC-SkMCs. CNN1 is highly expressed in smooth muscle, but can also be detected in skeletal muscle [37] (Supplementary Figure S1G). All together, these results demonstrate the specificity of our skeletal muscle differentiation method and confirm what we and others previously reported [6,15]. As previously described by Caron et al. the remaining cells that do not stain positive for MF20 in the myotube culture at D24 are unfused myogenic progenitors or myoblasts [6,38]. Lastly, to assess whether hESC-SkMCs were functionally responsive in vitro, we stimulated the cells with nicotine (3 mM) and performed calcium imaging. hESC-SkMCs were able to respond, and we observed calcium transients upon nicotine stimulation (Supplementary Figure S1H).

### 3.2. MyHC Expression in hESC-SkMC Was Enhanced by Testosterone, Follistatin, Cocktail of Myokines and the Combination Treatment

**Figure 2:**
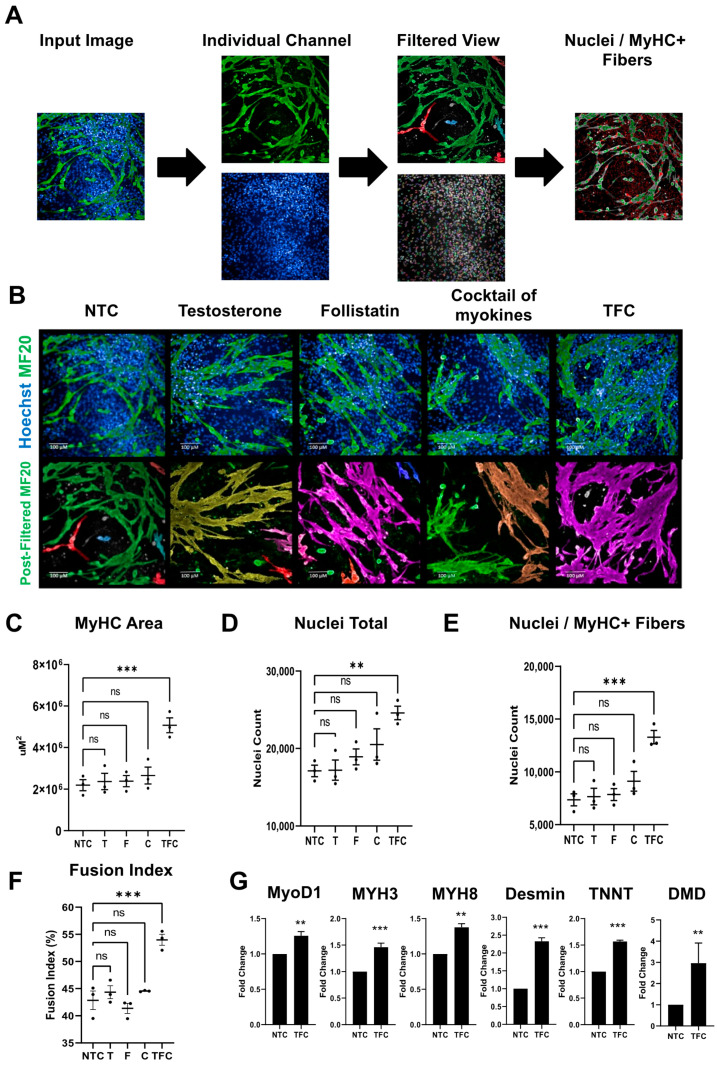
Anabolic factors and myokines enhance terminal differentiation of hESC-SkMC. (**A**) Image analysis pipeline. (**B**) Example pre- and post-filtered images of untreated and treated hESC-SkMC. Each color in the post-filtered MF20 images represents one segment of MF20 as determined by Harmony, the analysis software. Scale bar = 100 μm. (**C**–**F**) Image quantification of hESC-SkMCs in different treatments: MyHC area (**C**), total nuclei (**D**), nuclei within MyHC+ fibers (**E**) and fusion index (**F**). N = 3 for each condition. Results are the averages of four independent technical replicates over three independent experiments. Statistical analysis was performed using one-way ANOVA with Benjamini–Hochberg FDR correction. ** *p* < 0.01, *** *p* < 0.001. (**G**) TFC enhanced expression of several key myogenesis markers assessed by RT-qPCR. Statistical analysis was performed using a two–tailed *t*-test. ** *p* < 0.01, *** *p*< 0.001, ns: not significant.

To improve the final stage of our differentiation protocol, we selected factors known to have positive effects on skeletal muscle growth or strength. These included creatine, a non-protein nitrogenous compound known to increase skeletal muscle strength and performance [39,40], and myokines which are up-regulated by exercise [41,42] and regulate muscle mass and function [43]. As the primary goal of our study was to improve the fusion of hESC-derived SkMCs, we chose myokines with reportedly direct effects on myoblast fusion—IL-4, IL-6 (interleukin-4 and 6) and vascular endothelial growth factor (VEGF) [44,45,46]; or myoblast differentiation and muscle regeneration—brain-derived neurotrophic factor (BDNF) [47]. For each of these factors, we chose the lower range of concentrations that are commonly used in the literature. Initially tested individually, each of these compounds had little or no detectable effect on hESC-SkMCs (data not shown). However, we noticed slight changes in myotubes’ morphology when these five compounds were added together. We therefore used these compounds as a mixture that we refer to as the “cocktail of myokines” (C) in this study. Additionally, based on their strong anabolic effects, we also selected the well-known steroid hormone testosterone (T) and the myostatin inhibitor follistatin (F) [48] as potential muscle differentiation enhancers. We assessed the effects of T, F, C and the combined treatment (TFC) on hESC-SkMCs. Brightfield images showed that the average myotube size was greater following treatment, indicating improved terminal differentiation and fusion capacity (Supplementary Figure S1I). We next performed a detailed morphological analysis on treated and non-treated hESC-SkMCs. Since myosin is the most abundant protein in muscle, and the expression level of this protein reflects skeletal muscle size and strength [49], we investigated myosin abundance in hESC-SkMCs following each treatment. MyHC expression was detected by immunofluorescence using the MF20 antibody (recognizing all MyHC isoforms) in hESC-SkMCs 96 h after treatment with T, F, C, TFC or vehicle control. Sizes of MyHC+ areas were analyzed using the Perkin Elmer Opera Phenix^TM^ automated high-content screening system that produces high-throughput imaging and image quantification. Briefly, input images were split into individual channels and a threshold filter was applied to remove background noise. Signals that passed the threshold were subsequently quantified (Figure 2A). While individual treatments had no significant effects on MyHC+ areas when compared to non-treated cells (NTC) (Figure 2B,C), MyHC+ area was significantly increased in TFC-treated cells (Figure 2C). MyHC+ myotubes were also thicker in TFC-treated cultures compared to NTC (Figure 2B,C). As myotubes are multinucleated, we quantified the number of nuclei within MyHC+ fiber and observed a significant increase in cell number in cultures treated with TFC (Figure 2E), which was associated with an increase in the number of nuclei (Figure 2D). In addition, we observed a significant increase in myotube fusion index (calculated as the number of nuclei within MyHC+ area divided by the number of total nuclei) after treatment with TFC (Figure 2F), demonstrating these cells had a better fusion capacity. To assess consistency of this treatment, we also evaluated TFC treatment on three additional hPSC lines. We observed similar results in a hiPSC line (ATCC-hiPSC) and a hESC line (GENEA002), and a similar trend in one other hESC line (H9 (WA09)) (Supplementary Figure S2A–C). RNAseq analysis also revealed that TFC treatment did not affect the purity of our hESC-SkMCs (Supplementary Figure S2D).

To evaluate the general effect of TFC on hESC-SkMC differentiation, we analyzed the expression levels of various skeletal muscle specific genes by RT-qPCR and found TFC enhanced the expression of MyoD1 (myoblast determination protein 1); MYH3 (embryonic) and MYH8 (perinatal) myosin heavy chain isoforms; desmin (muscle specific intermediate filament); and the sarcomeric structural proteins TNNT (troponin T) and DMD (dystrophin) (Figure 2G). We also compared their responsiveness to nicotine stimulation (Supplementary Figure S3A). Although no difference was observed in the mean amplitude of calcium transient between NTC and TFC-treated hESC-SkMCs (Supplementary Figure S3B), TFC-treated myotubes were more responsive to nicotine stimulation compared to NTC in both cell number and total surface area (Supplementary Figure S3C,D). Lastly, the TGFβ signaling pathway has been shown to be an important regulator of myoblasts’ differentiation, and its inhibition was reported to enhance skeletal muscle fusion efficiency in both primary SkMCs and hPSC-SkMCs [50,51]. We therefore compared the effect of TFC with that of the highly selective TGFβ inhibitor ITD-1 on hESC-SkMCs differentiation. In our system, we observed no significant increase in MyHC+ area or fusion index in cells treated with ITD-1, whereas TFC led to significant increases in MyHC+ area and fusion index (Supplementary Figure S3E,F). Collectively, these results demonstrate that the addition of TFC can further enhance terminal differentiation by promoting fusion and MyHC expression in hESC-SkMCs.

### 3.3. Skeletal Muscle Genes’ Expression Levels in hESC-SkMCs Were Slightly Enhanced by TFC Treatment

**Figure 3:**
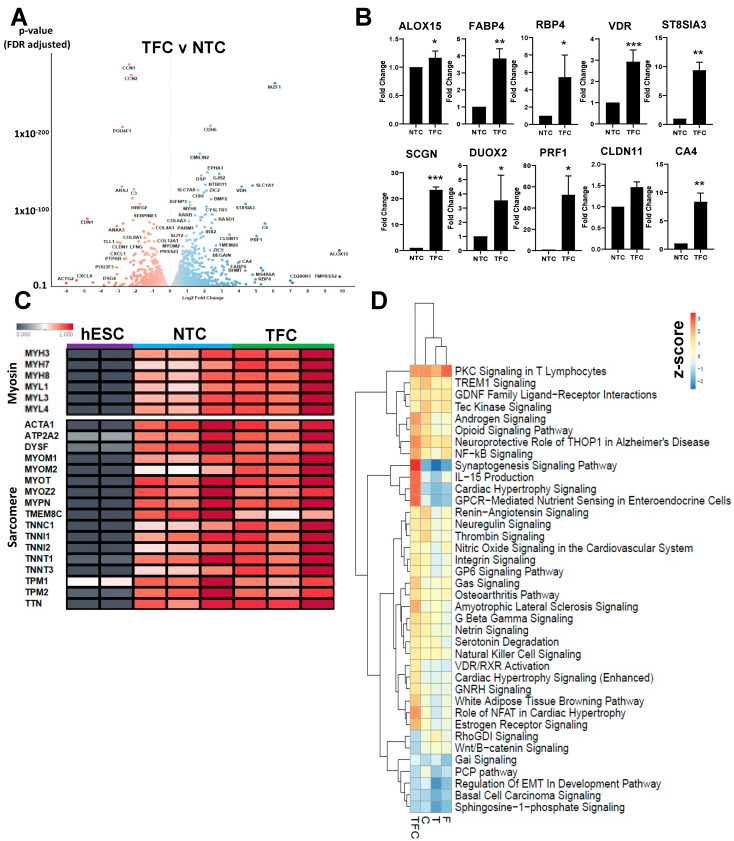
Transcriptomic profiling of NTC and TFC-treated hESC-SkMC. (**A**) Volcano plot of differentially expressed genes between TFC and NTC. (**B**) RT-qPCR validation of several top differentially expressed genes as identified via RNASeq. Statistical analysis was performed using a two-tailed *t*-test. * *p* < 0.05, ** *p* < 0.01, *** *p*< 0.001. (**C**) Heatmap comparison of various myosin and sarcomere genes between hESC, NTC and TFC-treated hESC-SkMC. (**D**) IPA comparative analysis of differentially regulated pathways in treated hESC-SkMCs compared to NTC. Shown are data pooled from three independent biological replicates.

To determine the gene expression changes behind enhanced MyHC expression and skeletal muscle fusion, we performed RNASeq to assess the transcriptomic profiles of NTC and treated hESC-SkMCs (T/F/C/TFC). Three biological replicates corresponding to hESC-SkMCs derived from three independent differentiation experiments were used for each of the treatments. Using padj < 0.05 and Log2FC > 1 as the thresholds, we observed 671 differentially expressed genes (DEG) in TFC treatment, including 471 up-regulated and 200 down-regulated genes compared to NTC (Figure 3A, Supplementary File S3). Surprisingly, many of these DEGs observed in TFC were different from those in individual treatments (T, F or C) (Supplementary Figure S3G,H, Supplementary File S4). To validate this transcriptomic profile, we performed RT-qPCR and confirmed the up-regulation of several top DEGs in TFC-treated hESC-SkMCs that we chose based on their reported roles in muscle development and function (Figure 3B). These genes are involved in a variety of biological processes known to be associated with skeletal muscle functioning, including metabolism of lipids (ALOX15, FABP4), interactions with vitamins (RBP4, VDR) and calcium binding (SCGN, DUOX2). Since TFC-treated hESC-SkMCs had overall higher MyHC protein expression and increased expression of MYH3 and MYH8 (Figure 2B,G), we next compared the expression levels of a number of myosin or sarcomere genes between NTC and TFC-treated hESC-SkMCs. Surprisingly, we did not observe any major differences (Figure 3C). Moreover, TFC-treated cells had minimal levels of MYH1 and MYH2 expression, suggesting treatment does not promote maturation towards adult MyHC (Supplementary Figure S3I).

### 3.4. Differentially Regulated Pathways Show Common and Divergent Patterns

Since transcriptional expression of skeletal muscle genes (Figure 3C) was not markedly enhanced by TFC treatment, we decided to identify the molecular pathways that were induced by TFC treatment. We compared the expression profiles between treated hESC-SkMCs (T/F/C/TFC) and NTC using IPA (Log2FC > 1, padj < 0.05) (Figure 3D, Supplementary File S5). DEGs were grouped into 222 pathways, and we observed pathways that shared similar expression trends in all treatments, such as PKC signaling and androgen signaling. Interestingly, TFC also up-regulated several pathways that were not up-regulated by individual treatments, such as cardiac hypertrophy signaling. Cardiac hypertrophy signaling is involved in sarcomere formation and includes the well-known myocyte enhancer MEF2C, which plays an important role in myogenesis [52].

### 3.5. TFC Greatly Enhanced Myosin and Sarcomere Gene Expression at the Protein Level

**Figure 4:**
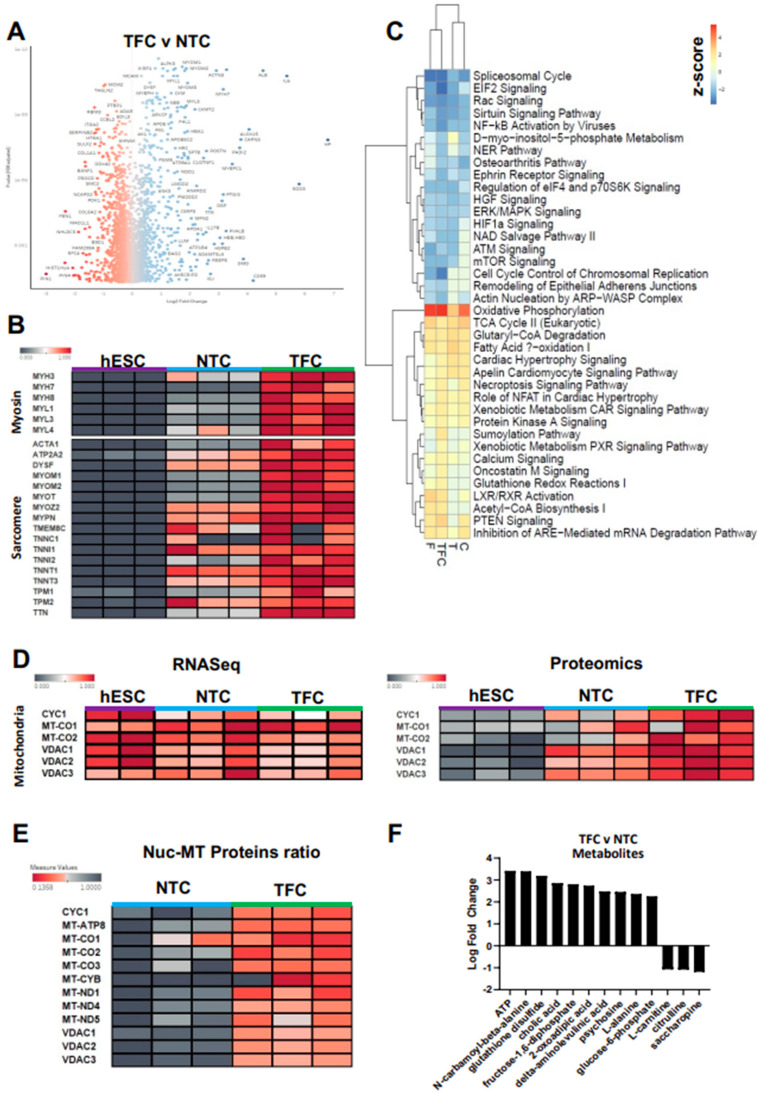
Proteomic profiles of NTC and TFC-treated hESC-SkMCs. (**A**) Volcano plot of differentially expressed proteins between TFC and NTC. (**B**) Heatmap comparison of various myosin and sarcomere proteins among hESC, NTC and TFC-treated hESC-SkMCs. (**C**) IPA comparative analysis of differentially regulated pathways in treated hESC-SkMCs compared to NTC. (**D**) Heatmap comparison of various mitochondrial genes at RNA (left panel) and proteins (right panel) levels among hESC, NTC and TFC-treated hESC-SkMC. (**E**) Heatmap comparison of nuclear (Histone H4) vs. mitochondrial protein ratios in NTC and TFC-treated SkMCs. (**F**) IPA metabolomic analysis (Log2FC > 1, padj < 0.05) of differentially expressed metabolites between TFC-treated cells and NTC. Shown are data pooled from three independent biological replicates.

Myogenesis is closely associated with a high level of post-transcriptional modification events [53,54,55]. Since we did not observe major changes in skeletal muscle-specific genes at the transcript level, we considered that differential expression between NTC and TFC might be more noticeable at the protein level. We therefore performed proteomics and compared NTC and treated hESC-SkMCs (T/F/C/TFC). We identified a large number of proteins (T = 195, F = 260, C = 214) that were not expressed in NTC but were expressed upon individual compound treatment (Supplementary Figure S4A, Supplementary File S6). Interestingly, a subset of 80 proteins were up-regulated in all three individual treatments. Gene Ontology pathway analysis showed these 80 proteins are involved in mitochondrial translation (57.14%) and Complex I biogenesis (42.86%) (Supplementary Figure S4B), suggesting these individual treatments enhance cellular respiration rate and ATP production. Surprisingly, 1033 proteins were not expressed in NTC but were present in TFC-treated SkMCs (Supplementary File S7). Among these, 803 proteins were only detected after TFC treatment and not after individual treatment (Supplementary Figure S4C,D). Gene Ontology analysis revealed these 803 proteins to be involved in diverse cellular activities, including glutamine and L-alanine transport, multivesicular body organization, organelle biogenesis and maintenance and metabolism (Supplementary Figure S4E). Next, we compared the proteomic profiles between NTC and TFC-treated hESC-SkMCs (Figure 4A) and assessed differentially regulated pathways via GSEA (Supplementary Figure S4F,G, Supplementary File S8). We observed highly up-regulated protein clusters strongly associated with muscle contraction, skeletal muscle development and respiratory chain electron transport. In contrast, major down-regulated gene clusters included condensed chromosome and regulation of mRNA processing, suggesting TFC-treated hESC-SkMC might be less transcriptionally active than NTC.

To determine whether the proteomic expression follows the transcriptomic profile, we evaluated the expression levels of the skeletal muscle markers shown in Figure 3C. Interestingly, while transcriptomic data showed no significant differences in skeletal muscle markers’ expression between NTC and TFC, we observed significant changes in these markers at the protein level. Various myosin heavy and light chain isoforms (MYH3, MYH7, MYH8, MYL1, MYL3 and MYL4) and sarcomere structural proteins, such as Titin (TTN) and actin alpha 1 (ACTA1), were significantly up-regulated in TFC compared to NTC (Figure 4B). However, MYH1 and MYH2 (adult MyHC isoforms) proteins remained undetected after TFC treatment (Supplementary Figure S3I). Pluripotency (NANOG, POU5F1 and SOX2) and proliferative markers (MKI67) were not detected at the protein level in either NTC or TFC-treated cells, indicating both protocols lead to a post-mitotic cellular state (Supplementary Figure S5A).

### 3.6. Oxidative Phosphorylation Was the Most Up-Regulated Pathway in Treated hESC-SkMCs

To identify the molecular pathways associated with protein changes, we performed an IPA (Log2FC > 1, padj < 0.05) comparison of the expression profiles of the treated hESC-SkMCs (T/F/C/TFC) and NTC, and observed oxidative phosphorylation as the most up-regulated pathway in all treatments, suggesting these anabolic factors and myokines had a positive effect on enhancing cellular respiration in hESC-SkMCs (Figure 4C). In addition to oxidative phosphorylation, several other pathways known to be involved in energy metabolism, including the TCA cycle, fatty acid B-oxidation and acetyl-CoA biosynthesis were also up-regulated in all treatments compared to NTC (Figure 4C). It is known that hESCs primarily utilize glycolysis for energy production and switch to oxidative phosphorylation as they differentiate into specialized cell types [56]. We did not observe significant differences in key mitochondrial genes’ expression levels when comparing NTC and TFC-treated hESC-SkMCs with RNAseq (Figure 4D). However, mitochondrial proteins were highly enriched in hESC-SkMCs compared to hESCs. Notably, mitochondrial proteins’ expression levels were much higher in TFC-treated hESC-SkMCs than in untreated cells, suggesting that TFC promotes greater metabolic activity (Figure 4D). We further assessed the expression of a large number of mitochondria-related genes between conditions and observed noticeably increased expression in TFC-treated cells at the protein level but not the mRNA level (Supplementary Figure S6A). Furthermore, we performed a nuclear to mitochondrial protein ratio comparison by normalizing both to the histone H4 protein expression level. Normalized expression ratios demonstrated that treated hESC-SkMCs had a higher mitochondrial protein ratio compared to NTC, and the overall effect was greatest with TFC (Figure 4E and Supplementary Figure S6B). We next performed metabolomics to assess the metabolites produced by NTC and TFC. Differential analysis revealed ATP to be the most enriched metabolite in TFC-treated hESC-SkMCs compared to NTC, matching the proteomics data, and is accompanied by the up-regulation of metabolic pathways such as glycolysis, fatty acid activation and the FAT10 signaling pathway (IPA Log2FC > 1, padj < 0.05) (Figure 4F and Supplementary Figure S5B).

### 3.7. TFC Treatment Enhanced Oxidative Phosphorylation in hESC-SkMCs

**Figure 5:**
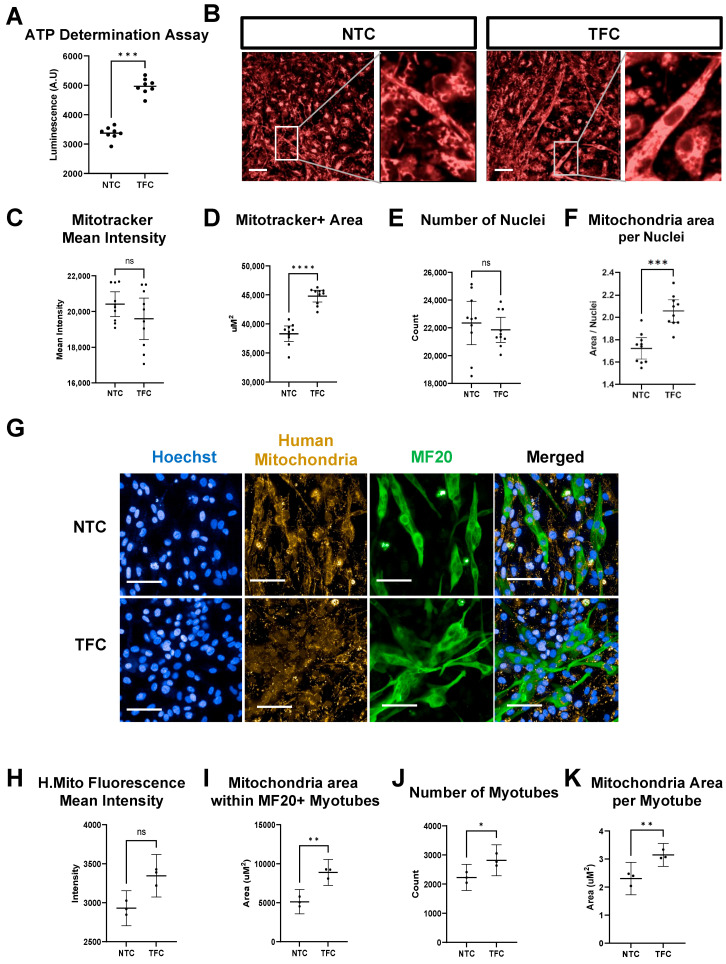
TFC treatment enhanced oxidative phosphorylation in hESC-SkMC. (**A**) Quantitative determination of ATP between NTC and TFC-treated hESC-SkMC. N = 8 for each condition. (**B**) Mitotracker signal in NTC and TFC-treated hESC-SkMC. Scale bar = 50 μm. (**C**–**F**) Quantification of Mitotracker measurement between NTC and TFC for Mitotracker signal intensity (**C**), Mitochondria area, determined by Mitotracker area (**D**), Number of nuclei (**E**) and Normalised mitochondria area per nuclei (**F**). One representative biological replicate with N = 10 technical replicates is shown for each condition. (**G**) Representative image of NTC and TFC-treated hESC-SkMC co-stained with human mitochondria antibody and MF20. Scale bar = 50 μm. (**H**–**K**) Quantification of mitochondria measurement between NTC and TFC for Mitochondria signal intensity (**H**), Mitochondria area within MF20+ myotubes (**I**), Number of myotubes (**J**) and Normalised mitochondria area per myotube (**K**). N = 3 independent biological replicates for each condition. Analysis performed with a two-tailed *t*-test. * *p* < 0.05, ** *p* < 0.01, *** *p* < 0.001, **** *p* < 0.0001, ns: not significant.

To confirm TFC treatment can enhance oxidative phosphorylation in hESC-SkMC, we performed quantitative ATP determination (Figure 5A). TFC-treated hESC-SkMCs had a significantly higher level of ATP compared to NTC (1.47 fold change). We next assessed mitochondrial mass in NTC and TFC-treated hESC-SkMCs using the fluorescent dye Mitotracker^TM^ Deep Red (250 nM) (Figure 5B–F). In skeletal muscle, mitochondria form a very dense network, making the visualization, counting and assessment of individual mitochondria difficult. Nonetheless, mitochondria in TFC-treated hESC-SkMCs appeared more elongated compared to those in the NTC, which were more often circular (Figure 5B). While we did not detect any major difference in Mitotracker signal intensity between NTC and TFC (Figure 5C), TFC-treated hESC-SkMCs had greater mitochondrial areas than NTC (Figure 5D), despite a similar number of nuclei per area (Figure 5E), with TFC-treated hESC-SkMCs showing a higher mitochondrial area per nucleus compared to NTC (Figure 5F). Similar results were obtained with a lower dose of Mitotracker (50 nM) (Supplementary Figure S7A–D).

To further confirm the results of Mitotracker measurement, we assessed mitochondrial content in myotubes by probing hESC-SkMCs with human mitochondria antibody together with MF20 (Figure 5G). TFC-treated myotubes showed a larger average mitochondria area compared to NTC, but no difference in mitochondrial signal intensity (Figure 5H,I). We normalized the size of mitochondrial area to the number of myotubes and demonstrated TFC-treated hESC-SkMCs to have a higher mitochondrial area per myotube compared to NTC (Figure 5J,K, Supplementary Figure S7E).

Collectively, our data demonstrate that TFC treatment enhanced the terminal differentiation and energy metabolism of hESC-SkMCs. We described here an improved skeletal muscle differentiation protocol leading to thicker muscle fibers and more numerous mitochondria.

## 4. Discussion

Muscle is a tissue with unique properties. It is capable of incorporating new nuclei into an already existing fiber in order to maintain its homeostasis. During human skeletal muscle generation and repair, proliferating myoblasts elongate and fuse with neighboring myoblasts, forming multinucleated and contractile myotubes [57]. This phenomenon requires a constant supply of new muscle cells, and therefore, the maintenance of an effective differentiation dynamic that is tightly regulated by growth factors and cytokines. Some of these factors, commonly termed myokines, are secreted by skeletal muscle (and also surrounding cells), and their roles in regulating muscle mass and strength have been extensively documented [43,58]. To date, over 600 myokines have been identified [43]. In this study, we selected several myokines (VEGF, IL4, IL6 and BDNF) with reported beneficial effects on skeletal muscle and tested their effects on hESC-SkMCs with or without the addition of the anabolic factors testosterone and follistatin. We report here that when combined together, testosterone, follistatin and the cocktail of myokines enhanced hESC-SkMC fusion and terminal differentiation.

We show that treatment with TFC enhanced the expression of several skeletal markers in hESC-SkMCs. Interestingly, multi-omics analysis revealed that TFC-treated hESC-SkMCs produced a distinct cellular profile compared to the hESC-SkMCs that received single treatments. These results suggest some of these DEGs may be regulated by multiple transcription factors, each being potentially activated by one of the different treatments (T, F or C). It is possible that the enhanced terminal differentiation and fusion capacity via TFC treatment is due to its ability to consistently promote the expression of these DEGs, which may not be possible with a single factor alone.

Importantly, while some muscle genes were only slightly up-regulated at the mRNA level following TFC treatment, proteomics analysis showed these genes were significantly up-regulated at the protein level, suggesting that the effect of TFC may occur through translational or post translational regulation. This observation is in accordance with numerous studies showing that skeletal muscle development and function are associated with high levels of translational regulation [55,59,60].

TFC-treated SkMCs also exhibited higher number of nuclei per fiber and a greater fusion index, suggesting these cells have a better capacity for fusion. This effect could at least partially be attributed to the myokine IL4, which has been shown to promote muscle growth by recruiting myoblasts and enhancing fusion [46]. TFC-treated SkMCs also had a higher total number of nuclei compared to NTC. However, cell cycle genes and proteins were not up-regulated in these cells following TFC treatment, and we did not observe enhanced proliferation in TFC-treated cells, suggesting that TFC does not induce cell proliferation. The higher number of nuclei was most likely due to a decrease in cell death during the differentiation/fusion process, reflecting the protective effect of myokines on skeletal muscle. In addition to increasing muscle capillarity through its angiogenic properties [61], VEGF also exerts a direct effect on skeletal muscle, and has been reported to promote the growth of myogenic fibers and protect myogenic cells from apoptosis [44]. In our RNAseq data, we found that three major apoptosis markers (CASP3, CASP7 and CASP9) had lower expression levels in TFC-treated hESC-SkMC compared to NTC. Statistically, CASP9 was significantly down-regulated (CASP3 Log2FC −0.12258, padj 0.33, CASP7 Log2FC −0.06119, padj 0.78, CASP9 Log2FC −0.24, padj 0.01). Most myokines have an anabolic effect and prevent muscle atrophy through a variety of biological processes. IL6 is a major myokine which, following exercise, is released by skeletal muscle to maintain its homeostasis [45]. IL6 plays a central role in skeletal muscle regeneration and hypertrophy by regulating satellite cell differentiation [62]. Similarly, muscle-secreted BDNF is essential to satellite cells activation and differentiation in response to muscle injury [47]. In this study, we also added creatine, a compound found primarily in skeletal muscle that can be produced endogenously or obtained through food consumption which provides rapid energy generation during skeletal muscle contraction and is known to induce skeletal muscle hypertrophy [63,64]. The combined positive effects of these molecules likely contributed to the better differentiation of treated SkMCs in this study. Surprisingly, we did not detect a significant effect of testosterone or follistatin on hESC-SkMC morphology or skeletal marker expression in the absence of the myokine cocktail, highlighting the essential role of these molecules. However, we still noticed a trend towards increased MyHC+ area in T- and F-treated hESC-SkMCs. Furthermore, both compounds are known to enhance energy metabolism in skeletal muscle [65,66]. To date, over 600 myokines have been reported, making it impractical to test all of them in our system in this current study. Further studies should assess the effects of other myokines on hESCs’ differentiation into SkMCs. Similarly, the concentrations used for TFC were supraphysiological in order to impart a timely response from the cells and compensate for the lack of many other factors in the medium. It is well-known that optimal concentrations of compounds vary greatly between hPSC lines [67]. Future studies could also evaluate different concentrations of T, F and C to optimize the effects of TFC in different hPSC cell lines.

Muscle contraction is mediated by the motor protein myosin, which binds to actin and drives filament sliding [68,69]. Different types of fibers exist in skeletal muscle, and they are characterized by the expression of specific MyHC ATPase isoforms. Generally, slow-twitch myofibers express the slow type I myosin ATPase and rely on oxidative metabolism. They have slow rate of contraction and are resistant to fatigue. In contrast, fast-twitch myofibers contract and fatigue rapidly, express fast type II MyHC and rely on glycolytic metabolism [70]. We previously showed by analyzing specific marker expression that our skeletal muscle differentiation protocol leads to the generation of both slow-twitch (TNNC1+ and TNNT1+) and fast-twitch (TNNI2+, TNNT3+, TNNC2+) myofibers from hPSCs [6]. In this new study, TFC treatment enhanced both slow and fast-twitch sarcomeric proteins level, which agrees with the metabolomic analysis, which showed that both fatty acid oxidation and glycolysis were increased. We also observed an increase in mitochondrial area in TFC-treated myotubes—a possible mechanism for their enhanced energy metabolism. Since proteomic data showed oxidative phosphorylation is the top up-regulated pathway in TFC-treated SkMCs, TFC may promote slow-twitch over fast-twitch myofibers due to the enhanced oxidative phosphorylation capacity.

Despite enhanced terminal differentiation following TFC treatment, these cells remained immature, as indicated by the presence of embryonic (MYH3) and neonatal (MYH8) markers and the lack of expression of adult myosin isoforms (MYH1 and MYH2). To date, maturation of hPSC-SkMCs to an adult stage can only be achieved to some extent in a 3D culture environment and might require the addition of other lineages, such as vascular cells and motorneurons [7,10,14]. Current 3D organoid cultures of hPSC-SkMC require extensive optimization of culture conditions to obtain uniform structures and may not be suitable for certain applications [71]. Future work will be focused on identifying factors that may lead to the generation of more mature, adult myotubes from hPSCs.

In humans, the MyHC level decreases with age [1], and a reduced level of MyHC subsequently leads to less muscle mass and weakened muscular function [72,73]. The identification of factors that enhance MyHC expression or myotube density in human skeletal muscle cells would in theory reinforce muscle contractile ability and delay the progression of muscle weakness. In this study, we report an improved protocol for skeletal muscle differentiation from hPSCs, based on increasing MyHC expression in hESC-SkMCs. This protocol is robust and can be employed at a large scale for drug screening applications. It may assist with future studies investigating human skeletal muscle development and identifying new therapies.

## Data Availability

The RNASeq data were deposited into the National Center for Biotechnology Information (NCBI) Gene Expression Omnibus (GEO) database with accession number GSE174144. Raw proteomics data have been deposited in the ProteomeXchange Consortium (http://proteomecentral.proteomexchange.org (accessed on 10 March 2022) via the PRIDE partner repository with the dataset identifier PXD025906, username: reviewer_pxd025906@ebi.ac.uk, password: fCoIAiDN. The datasets generated during and/or analyzed during the current study are available from the corresponding author on reasonable request.

## Supplementary Materials

The following supporting information can be downloaded at: https://www.mdpi.com/article/10.3390/cells11060963/s1, Figure S1: Immuno-staining of hESC differentiating into hESC-SkMC; Figure S2: TFC enhanced MyHC expression in hPSC lines including H9(WA09), GENEA002 and ATCC-hiPSC; Figure S3: TFC treatment increased number of responsive cells to calcium transient; Figure S4: Common and divergent gene expression between treated and untreated hESC-SkMC; Figure S5: TFC treatment increased ATP level in hESC-SkMC compared to untreated control; Figure S6: TFC treatment increased mitochondrial protein expression in hESC-SkMC compared to untreated control; Figure S7: Comparison of mitochondria abundance between NTC and TFC treated hESC-SkMC; Table S1: Table of Antibodies Used; Table S2: Table of Growth Factors Used; Table S3: RT-qPCR Primer Sequences; File S1: hESC-SkMC vs. hESC IPA Upstream Regulators; File S2: hESC-SkMC vs. hESC IPA Canonical Pathways; File S3: TFC vs. NTC treated hESC-SkMC RNASeq DESeq2 Analysis; File S4: All treatments vs. NTC treated hESC-SkMC RNASeq DESeq2 Analysis; File S5: All treatments vs. NTC treated hESC-SkMC RNASeq IPA Comparative Analysis; File S6: Individual treatment specific proteins list; File S7: TFC-treatment specific proteins list; File S8: GSEA of TFC vs. NTC treated hESC-SkMC.
