# Supplementary material for: Anabolic Factors and Myokines Improve Differentiation of Human Embryonic Stem Cell Derived Skeletal Muscle Cells
Source: Cells. 2022 Mar 11;11(6):963. doi: 10.3390/cells11060963 (PMC8946006; doi:10.3390/cells11060963)
Supplement: Supplementary file 1 [file cells-11-00963-s001.zip › cells-1608848-supplementary figures.pdf]

Supplemental. 1

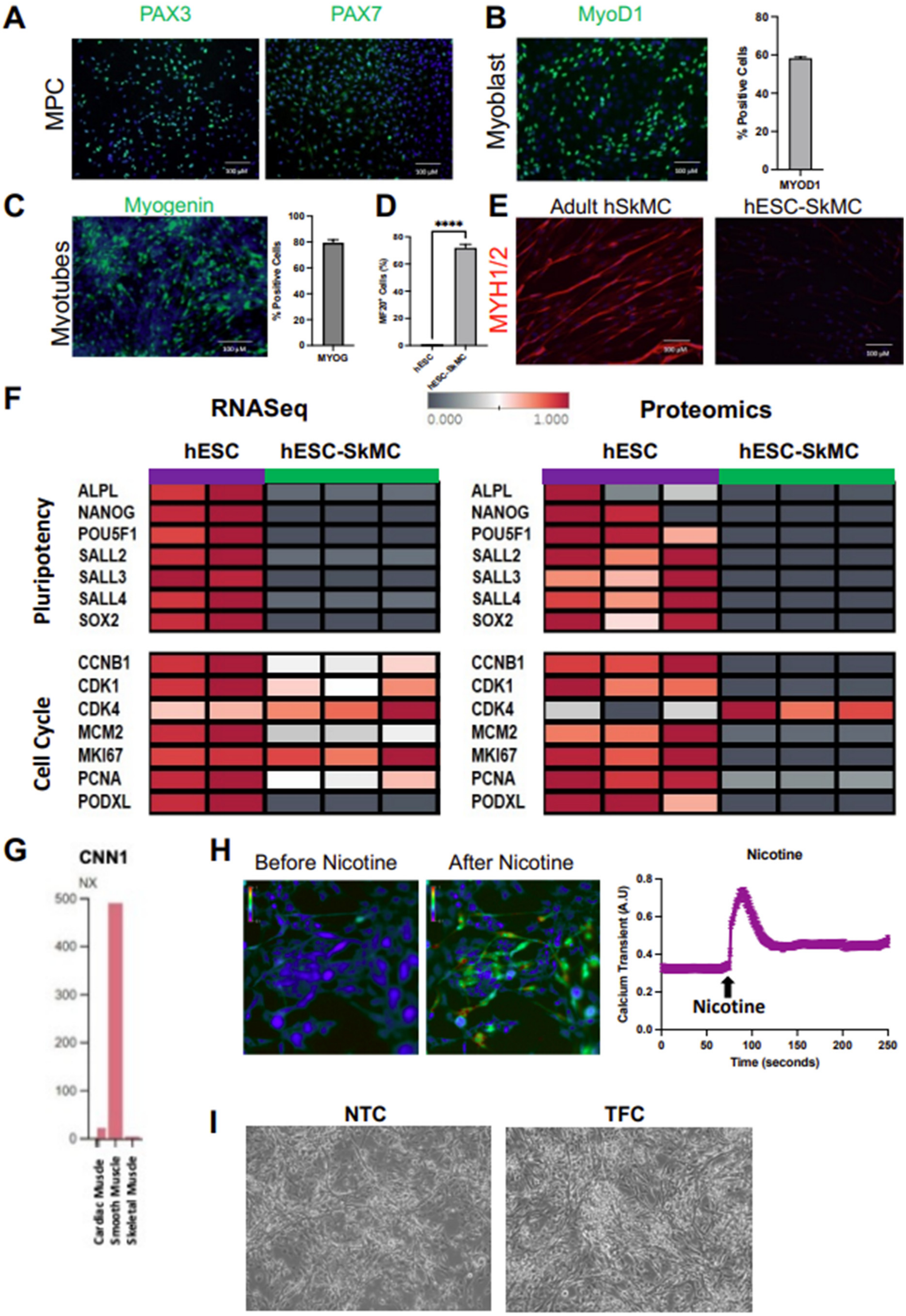

Figure S1. (A-C) Immuno-staining of cells with skeletal muscle markers during skeletal muscle differentiation.

PAX3 and PAX7 (A), MyoD1 (B) and MYOG (C). (D) Immuno-staining of adult primary SkMC and hESC-SkMC with 6H1 antibody (DSHB) marking both adult MyHC isoforms MYH1 and MYH2. (E) Skeletal muscle differentiation is associated with down-regulation of pluripotency and cell cycle genes. (F) CNN1 is expressed in all three types of muscle. Data obtained from The Human Protein Atlas website https://www.proteinatlas.org/ENSG00000130176-CNN1. (G) Representative image of calcium transients following the addition of nicotine. (H) Representative brightfield image of untreated and treated hESC-SkMC. Shown is one representative experiment of at least 3 biological replicates. MYOG was performed in three technical replicates.

Supplemental. 2

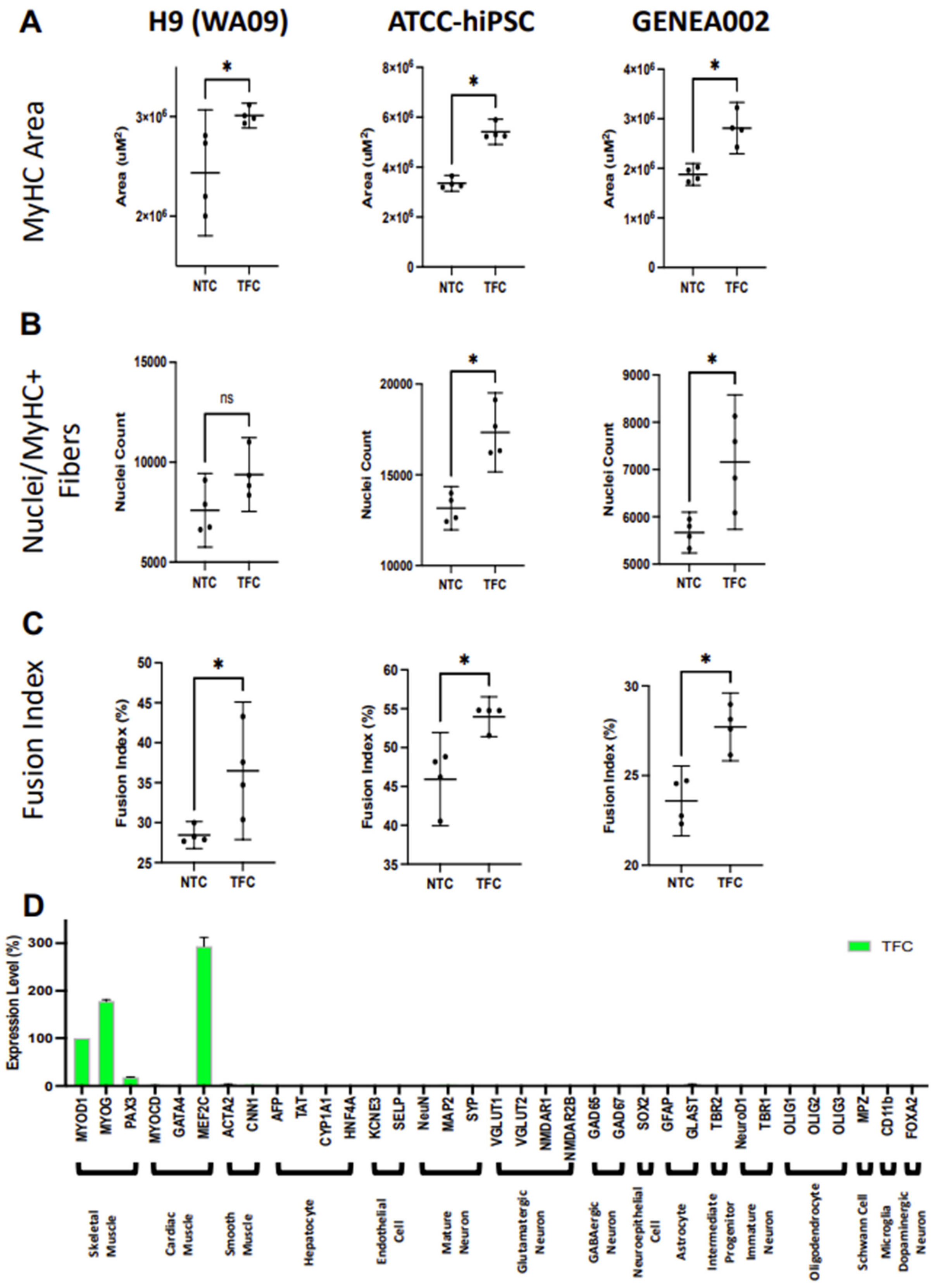

Figure S2. TFC enhanced MyHC expression in hPSC lines including H9 (WA09), GENEA002, and ATCC-hiPSC. (A) MyHC area. (B) Nuclei within MyHC+ fibers. (C) Fusion index. One representative biological replicate with N = 4 technical replicates is shown for each condition. Analysis performed with two tailed t-test. *P < 0.05, **P < 0.01,

***P< 0.001. (D) TFC treated hESC-SkMC express markers specific to skeletal muscle lineage. Shown is data pooled from 3 independent biological replicates.

Supplemental. 3

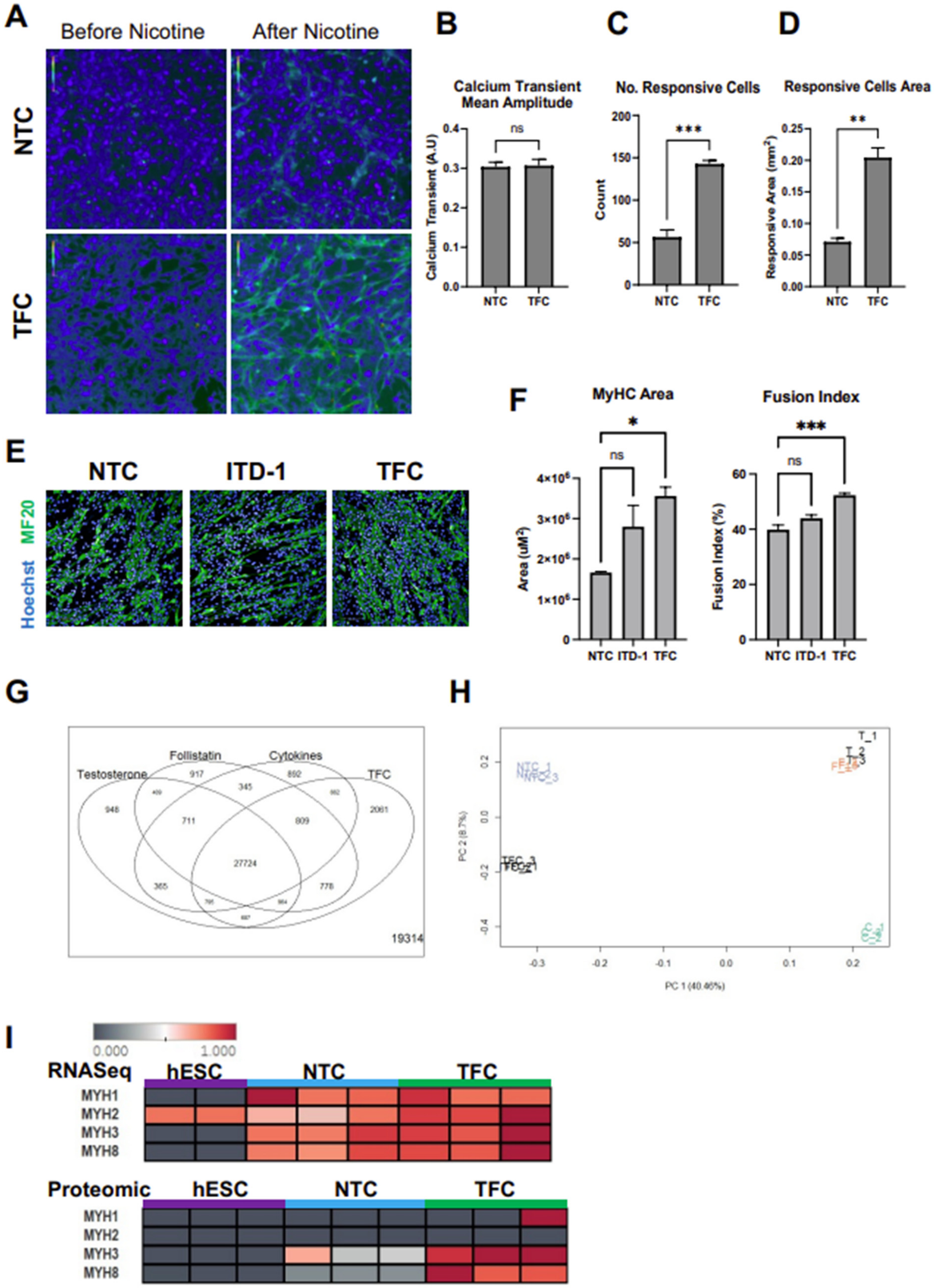

Figure S3. (A) Representative image of calcium transients following the addition of nicotine between NTC and TFC treated hESC-SkMC. (B) No difference in calcium transient mean amplitude between NTC and TFC. (C) More cells in TFC treated hESC-SkMC responded to nicotine stimulation compared to NTC. (D) Greater cellular area in TFC treated hESC-SkMC responded to nicotine stimulation compared to NTC. N = 3 for each condition. Analysis performed with two tailed t-test. *P < 0.05, **P < 0.01, ***P< 0.001. (E) Representative image of hESC-SkMC treated with ITD-1 (5μM) or TFC compared to control. (F) Quantification of MyHC area and fusion index between control, ITD-1 and TFC treated hESC-SkMC. Statistical analysis performed using One-way ANOVA with Benjamini-Hochberg FDR correction. *P < 0.05, **P < 0.01, ***P < 0.001. (G) Venn diagram showing common and divergent genes between different treatments (T, F, C and TFC) in RNASeq. (H) Principal Component Analysis (PCA) of untreated and treated hESC-SkMC in RNASeq. (I) NTC and TFC express high level of MYH3 and MYH8 and minimal level of MYH1 and MYH2.

Supplemental. 4

A

Condition	No. Proteins not detected in NTC
Testosterone	195
Follistatin	260
Cocktail of myokines	214
T and F	105
T and C	98
F and C	125
T and F and C	80

B

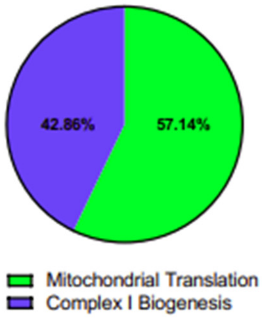

C

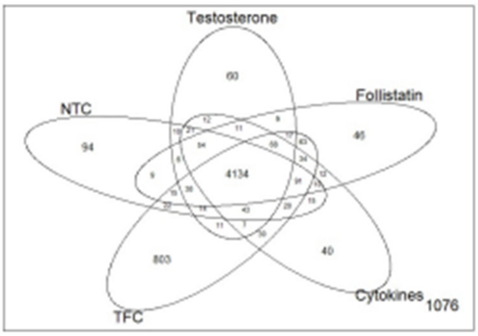

D

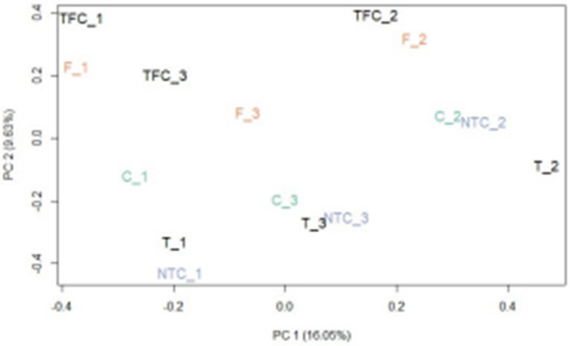

E

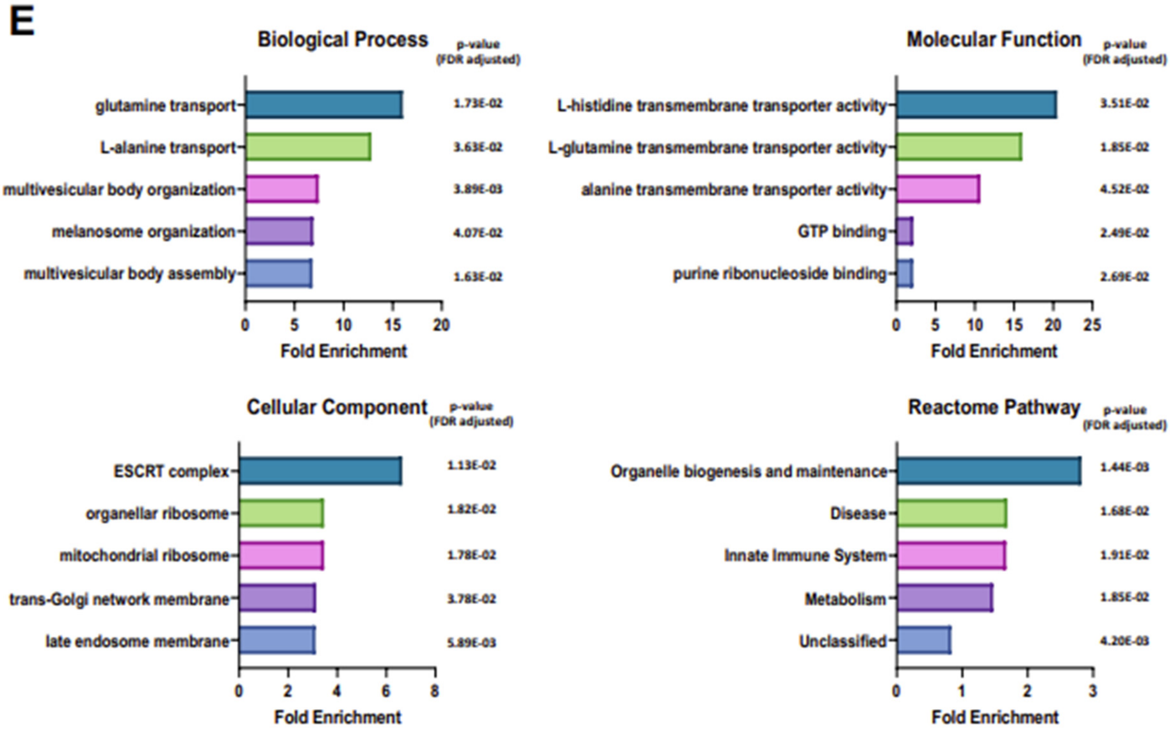

Supplemental. 4

F

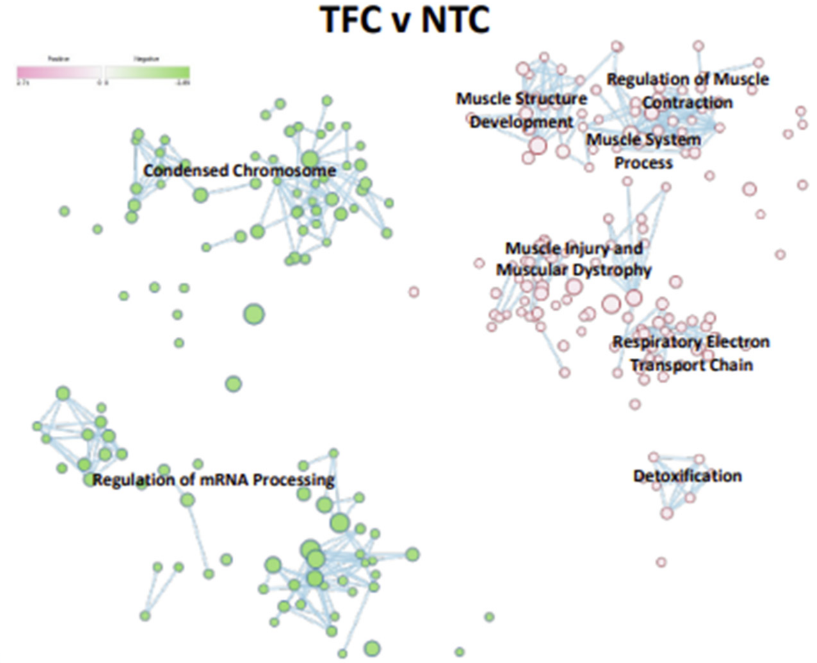

G

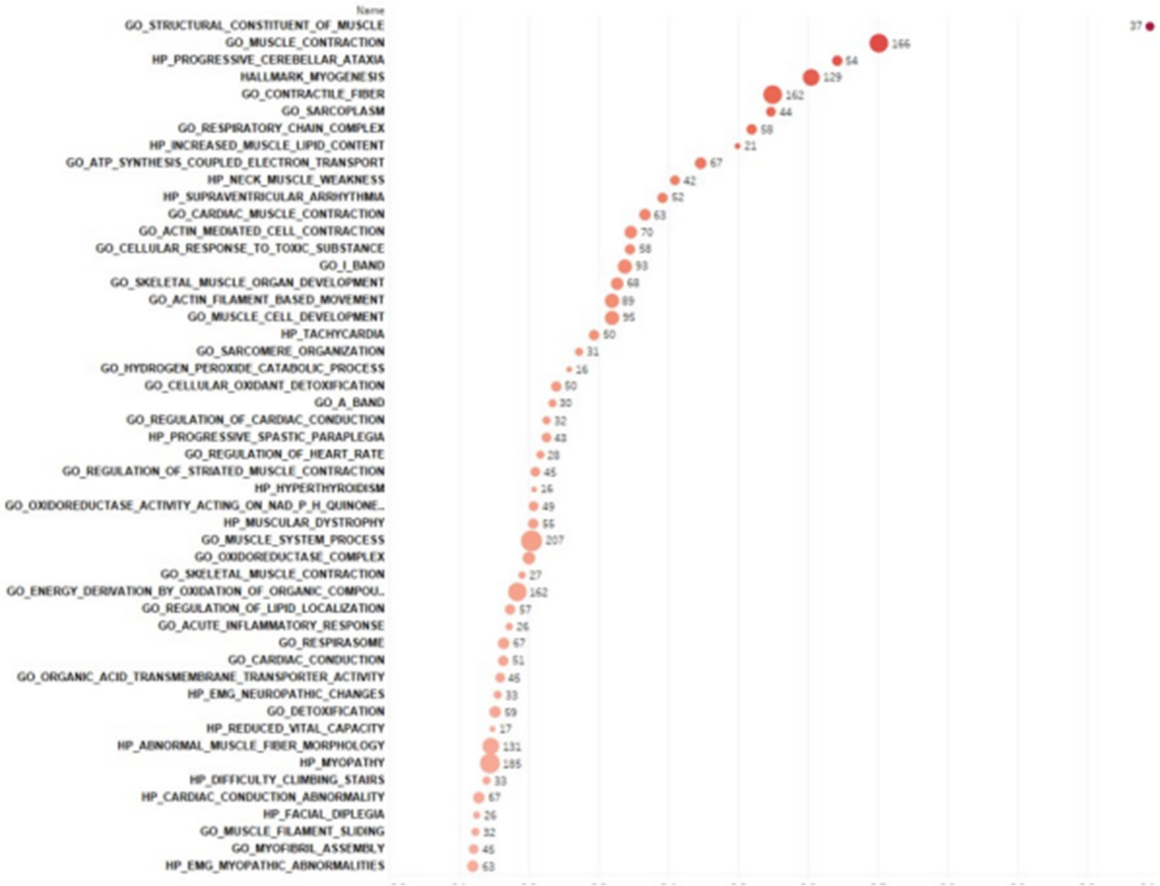

Figure S4. (A) Individual treatment induced expression of genes not detected in NTC in proteomics. (B) Gene Ontology analysis of the pathways associated with these 80 genes up-regulated in all three individual treatment. (C) Venn diagram showing common and divergent proteins between different treatments (T, F, C and TFC). (D) PCA of untreated and treated hESC-SkMC in proteomics. (E) Gene Ontology analysis of genes detected only in TFC treatment in proteomics. (F) GSEA analysis of major differentially regulated pathways between TFC and NTC in

proteomics. (G) GSEA analysis of top enriched gene sets in phenotype TFC compared to NTC. Shown is data pooled from three 3 independent biological replicates.

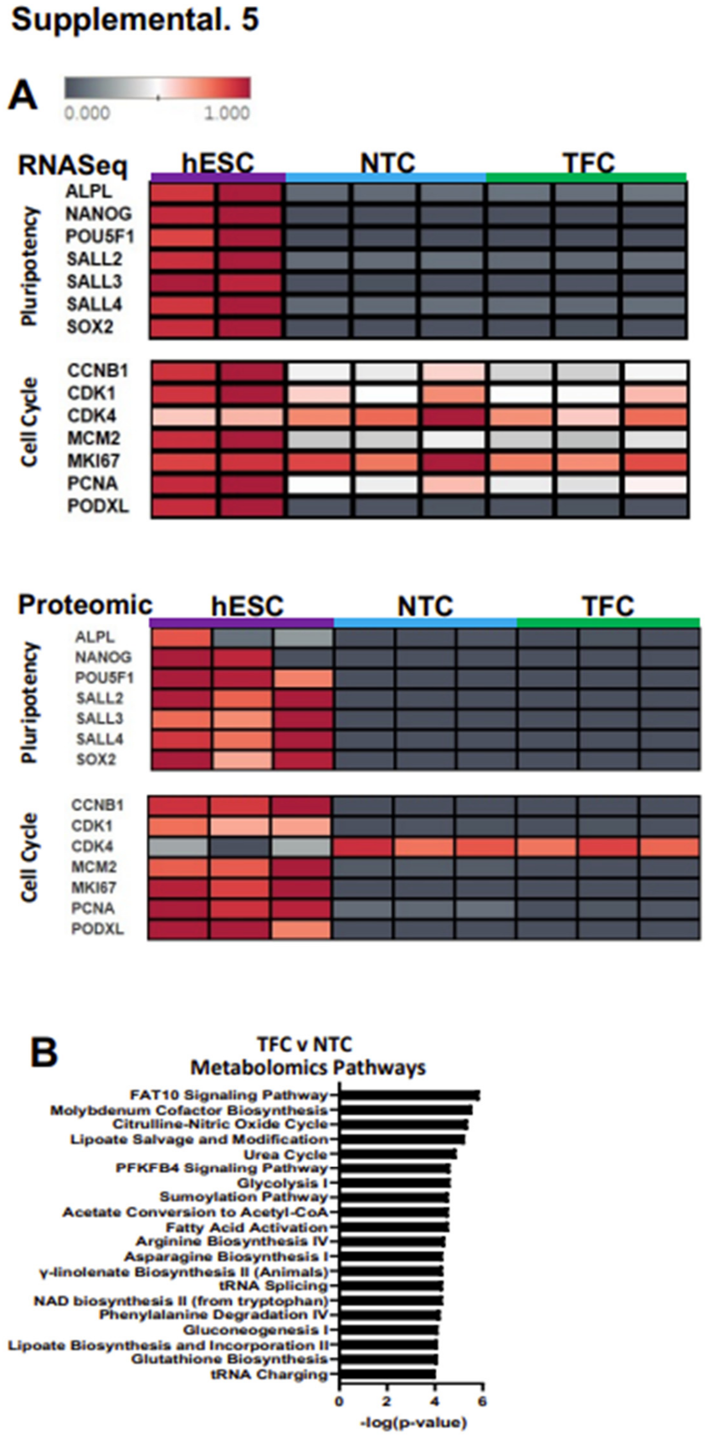

Figure S5. (A) Heatmap comparison of pluripotency and cell cycle markers between hESC, NTC and TFC. (B) Top enriched metabolomics pathways between TFC and NTC (IPA Log2FC>1, padj<0.05). Shown is data pooled from three independent biological replicates.

Supplemental. 6

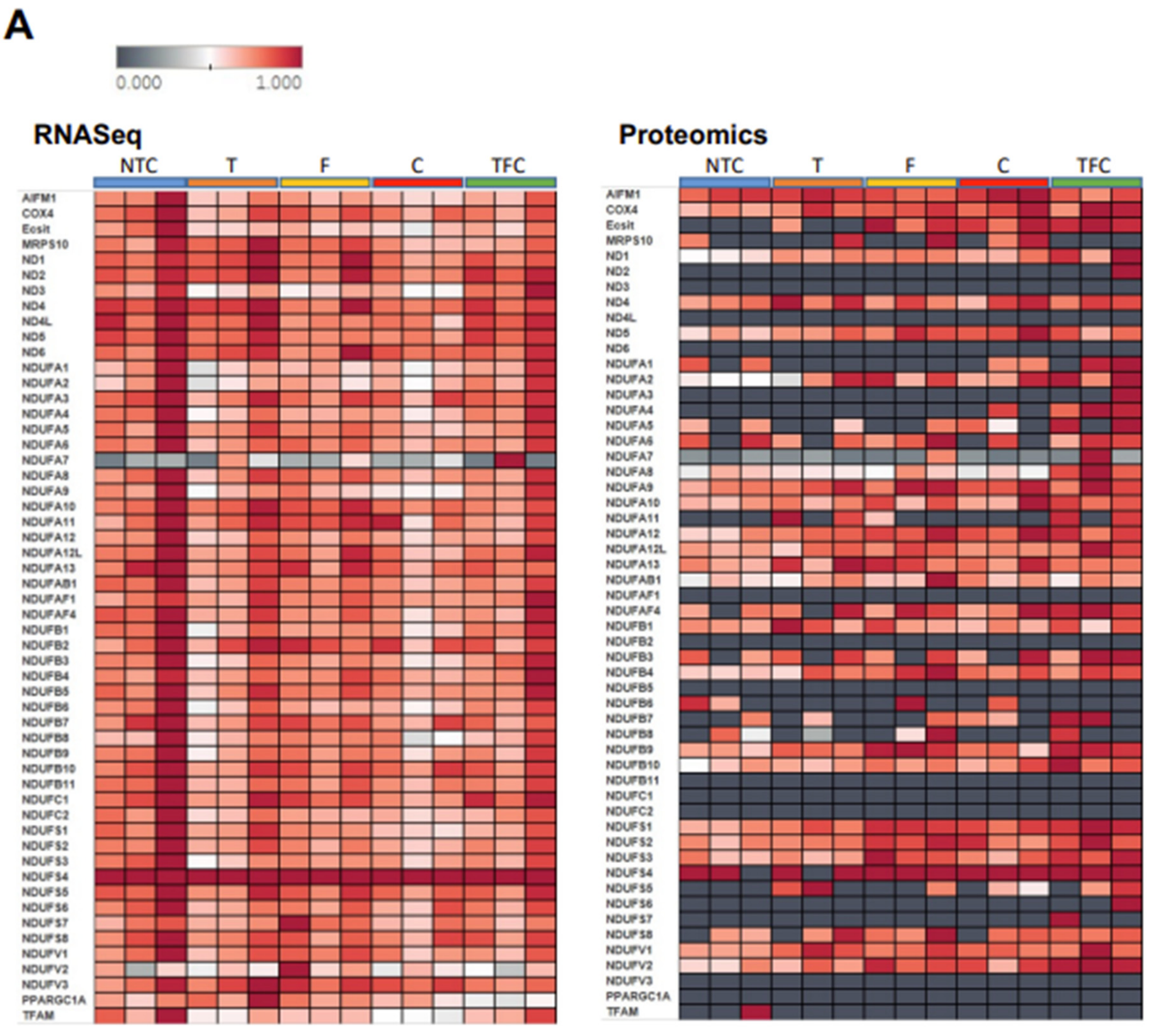

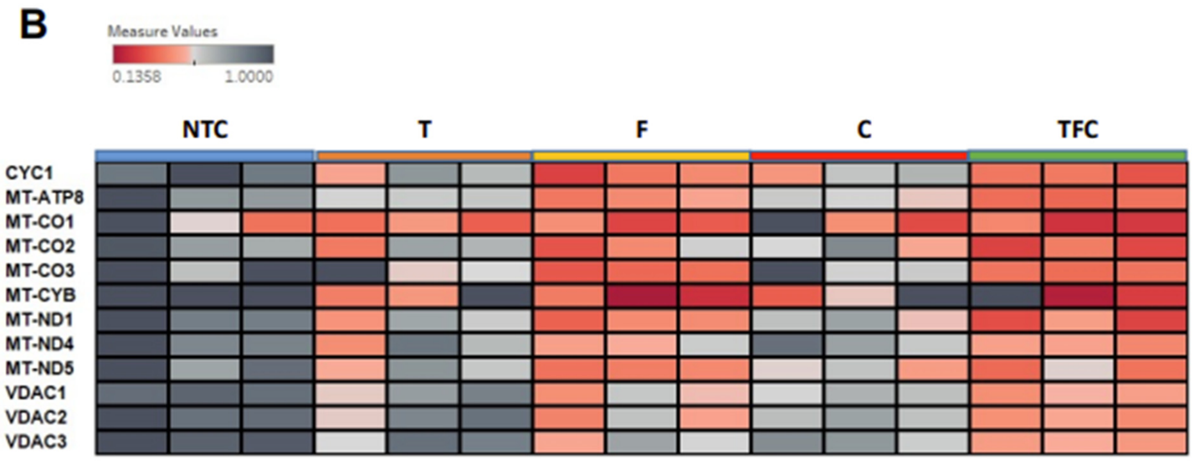

Figure S6. (A) Heatmap showing the expression of various mitochondrial genes between treatments at the RNA and protein level. (B) Heatmap comparison of nuclear (Histone H4) vs mitochondrial protein ratio in untreated (NTC) and treated hESC-SkMC. Shown is data from three independent biological replicates.

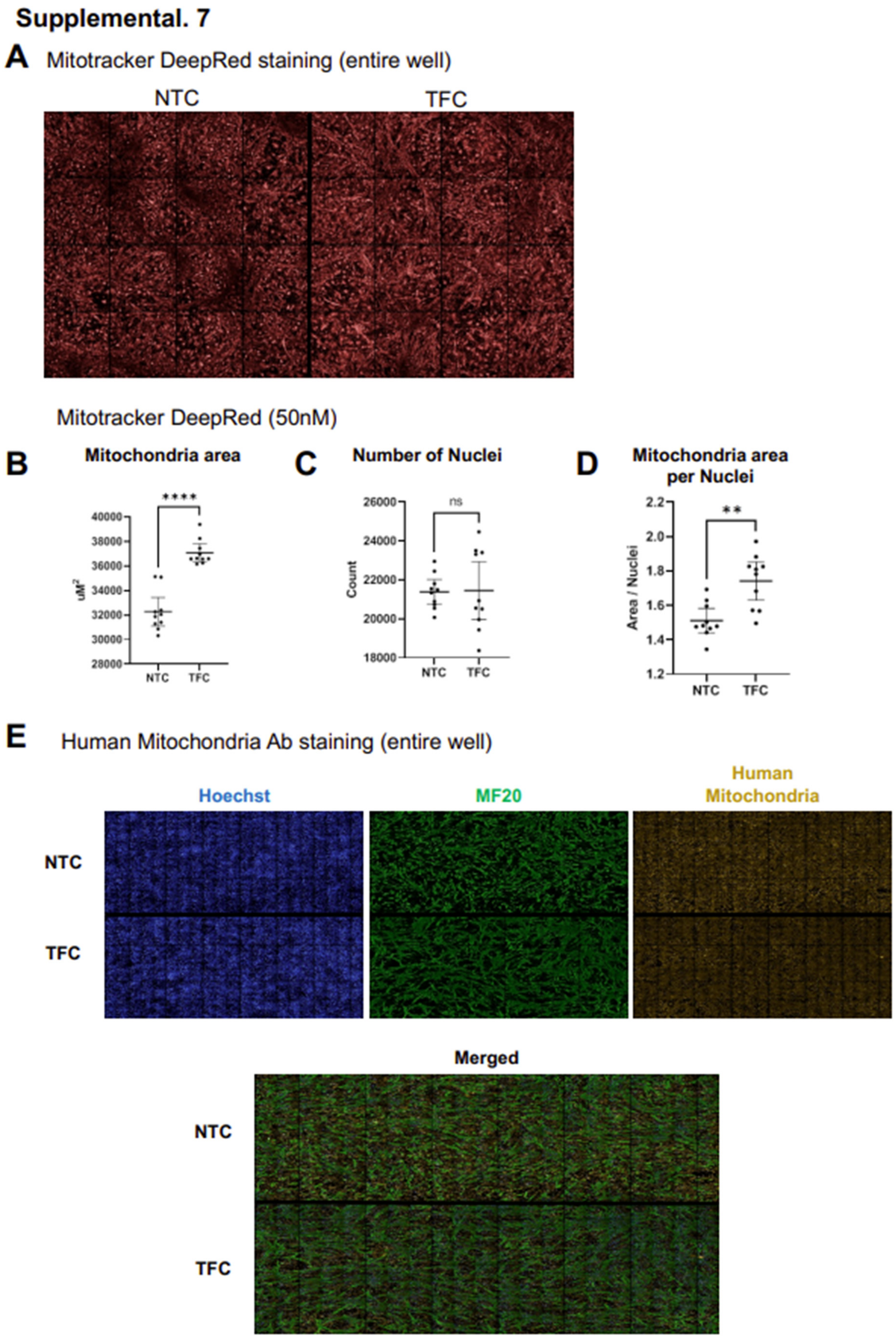

Figure S7. Comparison of mitochondria abundance between NTC and TFC treated hESC-SkMC. (A) Full well view of a representative Mitotracker staining of NTC and TFC treated hESC-SkMC. (B-D) Quantification of mitochondria

area between NTC and TFC treated hESC-SkMC using 50nM of Mitotracker Deep Red for area (B), Number of nuclei (C), and Normalised mitochondria area per nuclei (D). One representative biological replicate with N = 10 technical replicate is shown for each condition. Analysis performed with two tailed t-test. *P < 0.05, **P < 0.01, ***P< 0.001. (E) Representative image of human mitochondria antibody co-stained with MF20 in NTC and TFC treated hESC-SkMC.
